# Individual-based primary prevention of cardiovascular disease in Cambodia and Mongolia: early identification and management of hypertension and diabetes mellitus

**DOI:** 10.1186/1471-2458-12-254

**Published:** 2012-04-02

**Authors:** Dugee Otgontuya, Sophal Oum, Enkhtuya Palam, Manju Rani, Brian S Buckley

**Affiliations:** 1 Public Health Institute, Ministry of Health, Ulaanbaatar, Mongolia; 2 University of Health Sciences, 73 Monivong Blvd, Phnom Penh, Cambodia; 3 Western Pacific Regional Office, World Health Organization, Corner Taft and UN Avenue, Manila 1000, Philippines; 4 Department of Surgery, Philippine General Hospital, University of the Philippines Manila, Philippines

## Abstract

**Background:**

To assess the coverage of individual-based primary prevention strategies for cardiovascular disease (CVD) in Cambodia and Mongolia: specifically the early identification of hypertension and diabetes mellitus, major proximate physiological CVD risk factors, and management with pharmaceutical and lifestyle advice interventions.

**Methods:**

Analysis of data collected in national cross-sectional STEPS surveys in 2009 (Mongolia) and 2010 (Cambodia) involving participants aged 25-64 years: 5433 in Cambodia and 4539 in Mongolia.

**Results:**

Mongolia has higher prevalence of CVD risk factors than Cambodia --hypertension (36.5% versus 12.3%), diabetes (6.3% versus 3.1%), hypercholesterolemia (8.5% versus 3.2%), and overweight (52.5% versus 15.5%). The difference in tobacco smoking was less notable (32.1% versus 29.4%).

Coverage with prior testing for blood glucose in the priority age group 35-64 years remains limited (16.5% in Cambodia and 21.7% in Mongolia). Coverage is higher for hypertension. A large burden of both hypertension and diabetes remains unidentified at current strategies for early identification: only 45.4% (Cambodia) to 65.8% (Mongolia) of all hypertensives and 22.8% (Mongolia) to 50.3% (Cambodia) of all diabetics in the age group 35-64 years had been previously diagnosed.

Approximately half of all hypertensives and of all diabetics in both countries were untreated. 7.2% and 12.2% of total hypertensive population and 5.9% and 16.1% of total diabetic population in Cambodia and Mongolia, respectively, were untreated despite being previously diagnosed.

Only 24.1% and 28.6% of all hypertensives and 15.9% and 23.9% of all diabetics in Mongolia and Cambodia, respectively were adequately controlled. Estimates suggest deficits in delivery of important advice for lifestyle interventions.

**Conclusions:**

Multifaceted strategies are required to improve early identification, initiation of treatment and improving quality of treatment for common CVD risk factors. Periodic population-based surveys including questions on medical and treatment history and the context of testing and treatment can facilitate monitoring of individual-based prevention strategies.

## Background

Non-communicable diseases (NCD) account for a growing proportion of morbidity and premature mortality worldwide. This public health problem is neither exclusively nor predominantly associated with high-income countries (HIC). The total number of NCD-related deaths is proportionate to the distribution of populations, with almost 80% occurring in low- and middle-income countries (LMIC). But this apparent parity masks the disproportionate impact of NCDs in LMICs. For example, in 2008, the age-adjusted NCD mortality rate per 100,000 people was 757 in low-income countries compared with 380 in HICs [1].

Cardiovascular disease (CVD) accounts for almost half of all NCD-related deaths and is the leading cause of death in the developing world. In LMICs nearly 58% of CVD deaths are in those under the age of 60 compared with just 20% in HICs [2]. The increasing CVD burden largely results from the increased prevalence of modifiable risk factors such as tobacco use, unhealthy diets, limited physical activity, elevated blood cholesterol and glucose levels and hypertension, which in turn are associated with a range of inter-linked social trends: changing life-styles, dietary habits, occupations, increasing urbanization, globalization and demographic changes [3].

In recent decades, either as part of routine health care or of specific CVD prevention programs, developed countries achieved reduction in CVD morbidity and premature mortality through combinations of three strategies: population-level primary prevention strategies (intended to reduce the burden of CVD by reducing overall levels of modifiable risk factors in the whole population); individual-based primary prevention strategies (health service and advice provision intended to prevent the onset of CVD through risk factor management in individuals with established CVD risk factors but no disease); and individual-based secondary prevention strategies (health service provision and advice intended to prevent disease progression in people with established CVD). Research from several countries has consistently shown that reductions in risk factors explain more of the decline in CVD mortality than treatments of established disease. Between 42% and 60% of the decline in CVD deaths has been attributed to changes in risk factors including reduction in total cholesterol, systolic blood pressure and smoking prevalence, while 23% to 47% was attributed to treatments and secondary preventive therapies [4-9]. Population-based and individual-based primary prevention strategies can complement one another, both contributing to changes in risk factors. Their relative contribution is difficult to ascertain, however, and appears to vary in different settings. While in the US, significant decreases in systolic and diastolic blood pressure among patients with hypertension between 1988-94 and 2007-08 have been attributed to better medical control, changes in dietary habits were considered a more likely explanation in Finland [9,10]. Regional variations aside, population and individual changes in modifiable risk factors can result in relatively rapid declines in CVD mortality [11].

This paper assesses the coverage of individual-based primary prevention strategies for CVD. In particular it considers the early identification and management of hypertension and diabetes mellitus, which are both major proximate risk factors for CVD. Recent research has highlighted very different levels of medical management of specific CVD risk-factors around the world but little evidence comes from low-income countries [12,13]. In addition, there is little evidence on utilization of non-medical lifestyle interventions as part of targeted primary prevention strategies. Using nationally representative and comparable data from two LMICs in Asia, this study examines the prevalence of CVD risk factors and coverage of early detection and management of hypertension and diabetes mellitus through medical treatment, life-style modification interventions or both.

### Study context

Throughout Asia, demographic and social changes have resulted in an epidemiological transition, from a total disease burden dominated by infectious disease to one dominated by NCDs. In many LMICs, this has resulted in a dual burden of infections diseases and NCDs and in many health systems are under-resourced and already stretched [14,15]. Thus both the challenge presented by NCDs and individual countries' capacities to address these challenges vary.

Two LMICs at different stages of transition are considered in this study. Mongolia, one of the least densely populated countries in the world with a population of less than 3 million is a lower-middle-income country with a growing economy centered on agriculture and mining. Cambodia, a low-income country of more than 13 million people, remains largely agricultural and rural [15,16]. In 2008, NCDs were estimated to account for almost 53% of life years lost in Mongolia, and 31% in Cambodia [1].

## Methods

The study uses data from STEPS surveys (STEPwise Approach to Surveillance) conducted in Cambodia and Mongolia (Table 1). STEP surveys are similar to *National Health and Nutrition Examination surveys *in wide use in many developed countries such as USA, Japan and South Korea [17-19]. They are cross-sectional nationally-representative surveys that combine interviews and physical examinations and use standardized questionnaires and measurement protocols. Aggregated results of both surveys have been published previously by internal national bodies [20,21].

**Table 1:** Survey sample sizes, sex and age, response rates and measurement methods

	Cambodia	Mongolia
Date of data collection	Feb - Apr 2010	Oct-Dec 2009

*Response rate*		
Questionnaire	96.3%	95%
Physical measurements	94.2%	95%
Biochemical measurements	92.7%	95%^1^

Sample size aged (25-64)	5,433	4,539 ^2^

Male: % (95% CI)	48.6 (47.2 - 50.1)	50.3 (48.2 - 52.3)

Age in years: mean (SD)	40.2 (0.20)	39.7 (0.28)

Measurement of Blood pressure (measured three times in sitting position)	NISSEI Digital Blood Pressure Monitor (Model DS-500)	Omron Model 5 Automatic Blood Pressure Monitor

Measurement of fasting Blood sugar and cholesterol	Whole capillary blood taken from fingertips; dry chemical reagent strips & Accutrend GCT instruments	Same as in Cambodia

^1 ^Biochemical measurements were carried out only on one-third of total sampled population in the age group 25-64 years old in Mongolia, and response rate given here refers to this one-third of the sampled population

^2 ^In Mongolia, the sampled population also included 15-24 years old, but these were excluded for this analysis.

Each survey used multi-stage stratified cluster random sampling to select participants 25-64 years old (15-64 years in Mongolia). Sampled individuals were interviewed in person to elicit information on selected demographic and socio-economic characteristics, tobacco and alcohol use, dietary intake, physical activity, and diagnosis and treatment history for diabetes and hypertension. The health examination part comprised of measurements of height, weight, blood pressure, resting pulse rate, and collection of blood samples. Cholesterol and blood glucose were measured for the whole sample in Cambodia and for every third respondent in Mongolia in the age group 25-64 years. For testing of blood glucose, participants were instructed to fast for at least 12 hours and not to consume anything except plain water. Participants on anti-diabetic medications were advised to refrain from taking these medications on the morning of the test but bring the medications with them to take after the testing was completed. The participants were advised to take any other medications as usual. Table 1 summarizes the sample sizes, response rates and measurement methods. All sampled adults provided written informed consent and the surveys were approved by National Ethics Committee for Health Research of Ministry of Health in Cambodia (number 132 NECHR dated December 4, 2009) and by the Medical Ethical Committee of the Ministry of Health in Mongolia (Number 13, dated August.3, 2009) The survey methodology has been described in detail elsewhere [20-22].

### Outcome variables: definitions

The criteria by which prevalence is calculated for different NCD risk factors are presented in Table 2. We also report estimates of key service provision indicators--level of prior testing/diagnosis, and treatment as defined below.

**Table 2:** Criteria used to define and calculate prevalence of risk factors

Risk factor variable	Definition/numerator	Denominator
Diabetes prevalence	Respondents with survey-measured fasting blood glucose ≥ 6.1 mmol/l/110 mg/dl (whole blood); or measurement below threshold but respondent reporting insulin or other medication.	All respondents aged 25-64 with biochemical measurements at time of survey
Hypertension prevalence	Respondents with systolic blood pressure ≥ 140 mmHg or diastolic blood pressure ≥ 90 mmHg diastolic; or measurements below thresholds but respondent reporting anti-hypertensive medication.	All respondents aged 25-64 with measurement of blood pressure at the time of survey
Hypercholesterolemia prevalence	Measurement of total cholesterol ≥ 6.2 mmol/l (240 mg/dl) at the time of survey; or measurement below threshold but currently on medication for raised cholesterol.	All respondents aged 25-64 with measurement of blood cholesterol at the time of survey
Physically underactive	Physical inactivity assessed to be < 600 MET (metabolic equivalent) minutes per week as per the Global Physical Activity Questionnaire (GPAQ) scoring protocol[22,25,26].	All respondents aged 25-64 years
Overweight	Body Mass Index ≥ 25 kg/m^2.^	All aged 25-64 years with height and weight measured (pregnant women excluded)
Current smoking	Respondents reporting positively to question "do you currently smoke any tobacco product such as cigarettes, cigars, or pipes"	All respondents aged 25-64 years

### Screening coverage

This indicator provides an estimate of the proportion of the population that is currently being tested for hypertension or diabetes, whether opportunistic, or in response to specific symptoms suggestive of hypertension or diabetes, or as part of a systematic screening program. The indicator was calculated for 35-64 years old population, as this group corresponds more closely with the ages for which guidance suggests screening is most important [23,24]. The question asked to assess prior testing was "have you ever had your blood pressure [blood sugar] measured by a doctor or other health worker?"

### Diagnosis coverage

This indicator estimates the proportion of the hypertensive or diabetic population that is aware of their diagnosis. It reflects the net outcome of the coverage of screening and the degree to which this is targeted at high-risk individuals. The indicator is calculated as the proportion of total hypertensive (or diabetic) respondents as identified at the time of survey (using criteria and definition reported in Table 2) who reported positively to the question "have you ever been told by a doctor or other health worker that you have raised blood pressure or hypertension [blood sugar or diabetes]?".

### Treatment amongst hypertensives and diabetics

Treatment status was elicited by a question: "Are you *currently *receiving any of the following treatments/advice for high blood pressure [diabetes] prescribed by a doctor or other health worker?" Response categories included medical treatment - "drugs (medication) that you have taken in past two weeks" (two separate categories for diabetes: insulin and other drugs) - and four non-medical categories - "advice to reduce salt intake" ("Special prescribed diet" for diabetes), "advice or treatment to lose weight", "advice or treatment to stop smoking", "advice to start or do more exercise". The denominator for assessing the treatment coverage includes both total hypertensive and diabetic population identified at the time of survey (as per the prevalence criteria in Table 2) or those who self-reported a previous diagnosis. It was considered important to include participants who self-reported a previous diagnosis but who were not classified as hypertensive or diabetic by blood pressure or glucose levels or by medication. This was so that analyses could take into account previously diagnosed individuals whose raised blood pressure or blood was managed through non-medical methods. Estimates are reported for the following: not receiving any treatment (either untreated despite being previously diagnosed or untreated due to being undiagnosed prior to survey); currently treated (with the following subgroups: treated by drugs alone; treated by at least one of the non-medical treatments described above; treated or by both drugs and at least one of the non-medical treatments).

### Treatment quality deficits

The proportion of the hypertensive (diabetic) population that reported they were on medication and who had blood pressure (blood sugar) higher than the diagnostic threshold at the time of survey was considered an indicator of suboptimal treatment quality. Proportions are also estimated of those with either condition who are overweight, physically underactive or current or previous smokers but who have not received appropriate advice about weight loss, exercise or smoking cessation respectively.

### Statistical analysis

All the *a*nalyses were conducted using STATA Version 11. Percentages and 95% confidence intervals were calculated for categorical variables. Using the "svy:" function in STATA, all estimated were weighted to correct for age-sex variation between samples and target national populations.

## Results

### Prevalence of risk factors

Mongolia has significantly higher prevalence of the major risk factors than Cambodia--hypertension (36.5% versus 12.3%), diabetes (6.3% versus 3.1%), hypercholesterolemia (8.5% versus 3.2%), and overweight (52.5% versus 15.5%). The difference in tobacco smoking prevalence was less notable (32.1% in Mongolia compared to 29.4% in Cambodia). However, Mongolia has a lower proportion of physical underactive population than Cambodia (6.5% versus 9.0%) (Table 3).

**Table 3:** Prevalence of cardiovascular risk factors in population aged 25-64 in Cambodia and Mongolia

Risk factor	Cambodia		Mongolia		
	**Number included in analysis**	**Value** **[95% CI]**	**Number included in analysis**	**Value** **[95% CI]**	**P-value (chi^2 ^Pearson test)**

Current smoking (%)	5433	29.4 [27.4-31.3]	4539	32.1 [30.3-33.9]	0.04
Physically underactive (%)	5432	9.0 [7.8-10.1]	4470	6.5 [4.3-8.6]	0.04
Overweight (%)	5224	15.5 [14.0-17.0]	4501	52.5 [49.7-55.3]	0.00
Hypertension prevalence - survey measured (%)	5316	12.3 [11.2-13.5]	4502	36.5 [34.2-38.8]	0.00
Diabetes prevalence - survey measured (%)	5124	3.1 [2.5-3.7]	1470	6.3 [4.5-8.0]	0.00
Hypercholesterolemia prevalence%	5210	3.2 [2.6-3.9]	1538	8.5 [5.3-11.7]	0.00

### Screening gap

In both the countries, the proportion of individuals aged 35-64 who reported prior measurement of their blood pressure was substantially higher than for prior testing for blood glucose (Table 4). While almost three quarters of individuals reported previous measurement for blood pressure in Mongolia, about half of the respondents reported so in Cambodia. Similarly, the proportion of individuals whose blood glucose was previously measured was lower in Cambodia (16.5%) compared to 21.7% in Mongolia.

**Table 4:** Screening and diagnosis coverage for hypertension and diabetes in 35-64 years old population

	Cambodia		Mongolia	
	**Number included in analysis**	**Value (%)** **95% CI**	**Number included in analysis**	**Value (%)** **95% CI**

Hypertension				

Previously tested	3979	51.3 [48.6 - 54.0]	3014	75.9 [73.8 - 78.0]
Previously diagnosed	695	45.4 [40.3 - 50.4]	1357	65.8 [61.5 - 70.1]

Diabetes				

Previously tested	3979	16.5 [14.4 - 18.5]	3014	21.7 [18.8 - 24.6]
Previously diagnosed	173	50.3 [40.6 - 60.0]	67	22.8 [6.9 - 38.6]

### Diagnosis gap

Consistent with higher screening coverage, proportions of total hypertensive individuals who were previously diagnosed were much higher in Mongolia (65.8%) compared to Cambodia (45.4%). However, despite the lower screening coverage for blood sugar in Cambodia, a significantly higher proportion of total diabetic individuals in Cambodia reported being previously diagnosed (50.3%) than in Mongolia (22.8%), which may suggest better targeting of testing to high-risk individuals in Cambodia (Table 4).

### Management of hypertension and diabetes

Approximately half of the total hypertensive population in both countries, was untreated, though the proportion was higher in Cambodia (57.8%) than in Mongolia (47.2%) (Table 5). Of the total hypertensive population, 7.2% in Cambodia and 12.2% in Mongolia were untreated despite being previously diagnosed, 50.6% and 35.0% were untreated due to being undiagnosed. In Cambodia 13.8% and in Mongolia 17.0% of hypertensives received non-medical treatments alone while 22.9% and 21.3% received both medical and non-medical treatments. At 14.6% the proportion receiving medical treatment alone was much greater in Mongolia than in Cambodia (5.4%).

**Table 5:** Treatment among hypertensive and diabetic individuals aged 25-64 years in Cambodia and Mongolia

	Cambodia	Mongolia
	**Value (%)** **(95% CI)**	**Value (%)** **(95% CI)**

Total hypertensive sample (N)*	(871)	(1938)

i) *Not treated%*	57.8^†^	47.1^†^
previously undiagnosed	50.6 [45.9 - 55.3]	35.0 [31.1 - 38.8]
previously diagnosed	7.2 [5.3 - 9.1]	12.2 [9.7 - 14.6]

ii) *on treatment*	42.2^†^	52.9^†^
% only non-medical treatment	13.8 [11.1 - 16.5]	17.0 [13.9 - 20.1]
% non-medical & medical treatment	22.9 [19.2 - 26.7]	21.3 [18.6 - 24.0]
% only medical treatment	5.4 [3.5 - 7.3]	14.6 [10.3 - 18.8]

*iii) Controlled *	28.6 [24.7 - 32.5]	24.1 [21.0 - 27.2]

Total diabetic sample (N)*	(208)	(175)

i) *Not treated%*	52.0^†^	53.4^†^
previously un-diagnosed	46.1 [37.7 - 54.6]	37.2 [27.0 - 47.4]
previously diagnosed	5.9 [2.2 - 9.6]	16.1 [8.4 - 23.9]

ii) *on treatment*	48.0^†^	46.6^†^
% only non-medical treatment	9.9 [6.0 - 13.9]	25.8 [18.5 - 33.1]
% non-medical & medical treatment	30.6 [23.2 - 38.0]	20.1 [11.6 - 28.5]
% only medical treatment	7.5 [1.9 - 13.0]	0.7 [0.0 - 2.2]

*iii) % controlled*	23.9 [16.8 - 30.9]	15.9 [9.8 - 22.1]

* Includes both survey-measured and self-reported

^† ^Figures were rounded from five decimal points STATA outputs to one decimal point for the table. The sum of multiple rounded figures in the table may not exactly equal 100%.

Slightly more than half of the total diabetic population in each country reported being untreated: 52.0% in Cambodia and 53.3% in Mongolia. In Mongolia nearly a third of these (16.1/53.4)were untreated despite previous diagnosis. This was the case for only one eighth (5.9/52.0)of untreated diabetics in Cambodia. At 25.8% the proportion of those receiving non-medical treatment alone in Mongolia was markedly higher than in Cambodia (9.9%) (Table 5).

Only around a quarter (28.6% and 24.1%) of the total hypertensive population and less than a quarter (23.9% and 15.9%) of the total diabetic population in Cambodia and Mongolia, respectively, had blood pressure or blood sugar levels that were controlled and less than the diagnostic thresholds upon survey measurement, including those on current medication.

### Treatment quality deficits

More than two-thirds of hypertensive individuals receiving medications in Mongolia, and one-third in Cambodia had inadequately controlled blood pressure. Point estimates suggest deficits in delivery of key health advice to hypertensive individuals who are smokers, overweight or obese or physically underactive, but the small numbers included in analyses in many instances undermine the strength of these findings. Levels of uncontrolled blood glucose in diabetics on medication and poor advice levels among high-risk diabetic subgroups appear also to be of concern, but again low numbers of cases are available for analyses (Table 6).

**Table 6:** Specific treatment quality deficits in management of hypertension and diabetes

	Cambodia		Mongolia	
**Indicator**	**Number included in analysis**	**Value %** **(95% CI)**	**Number included in analysis**	**Value%** **(95% CI)**

Hypertension				

On medication but blood pressure uncontrolled	292	38.5 [31.7 - 45.2]	690	68.8 [64.0 - 73.5]
Smokers reporting no smoking cessation advice	89	34.7 [22.2 - 47.2]	282	54.8 [47.6 - 61.9]
Overweight and reporting no weight loss advice	141	38.9 [29.4 - 48.4]	761	57.9 [50.7 - 65.1]
Inactive and reporting no exercise advice	48	39.1 [21.9 - 56.2]	98	57.4 [44.8 - 69.9]

Diabetes				

On medication but blood sugar uncontrolled	77	58.1 [44.4 - 71.8]	18	61.9 [30.0 - 93.9]
Smokers reporting no smoking cessation advice	23	13.2 [-2.4 - 28.9]	29	50.5 [25.0 - 76.1]
Overweight and reporting no weight loss advice	40	18.2 [6.8 - 29.6]	81	40.2 [22.2 - 58.2]
Inactive and reporting no exercise advice	14	20.6 [-1.1 - 42.2]	10	30.0 [-4.6 - 64.6]

## Discussion

Using nationally representative data, this study assessed coverage of individual-based primary prevention strategies involving early detection and management of risk factors for CVD in two LMIC countries in Asia.

The study revealed key CVD risk factors already at alarming levels, with hypertension in particular emerging as a major public health concern. This high prevalence of risk factors warrants aggressive population-wide strategies such as public awareness campaigns and appropriate legislation and policy measures to alter societal norms relating to dietary salt intake, tobacco smoking and physical inactivity [27]. Individual-based primary prevention using medical and non-medical measures to reduce risk factors in high-risk persons also needs to be scaled-up to complement population-wide measures.

Timely detection of underlying risk factors, a critical component of the individual-based primary prevention strategies, emerged as a major challenge. No formal guidelines for universal, targeted or opportunistic screening for CVD risk factors existed at the time of the surveys in both countries, though both prioritize early detection of NCDs in current national policy and program documents [16,28-30].

The results suggest that an improved proportion of cases might be detected by targeted testing. For example, while in Cambodia only 16.5% of 35-64 year old population reported previous testing for blood sugar and yet 50.3% of total diabetic cases were previously diagnosed. Indeed, universal screening may not be feasible in many LMIC: evidence-based guidelines for opportunistic screening of high-risk population groups as identified based on epidemiological profile may be the first step, although poor overall health service utilization may limit coverage [31,32]. The frequent co-existence in individuals of several risk factors justifies strategies/guidelines for concurrent screening for multiple risk factors, an approach that also allows assessment of the absolute global risk of developing CVD in the next five to ten years--the recommended approach for making treatment decisions as discussed later [33].

The treatment gap in each country comprises those who are untreated because they are as yet undiagnosed, plus the proportion of individuals who are untreated despite previous diagnosis. In the absence of any follow-up questions, it is hard to identify the reasons for lack of treatment in these previously diagnosed hypertensives and diabetics, which may be both supply and demand related: due to factors related to difficulties in accessing or paying for treatment, and in some cases personal attitudes or preferences. A substantial treatment gap amongst the previously diagnosed population suggests that any plans to improve early diagnosis must be accompanied by increased provision, accessibility and affordability of treatment.

Approximately 14% and 17% of total of population with raised blood pressure and 10% and 26% of diabetics in Cambodia and Mongolia, respectively, reported receiving non-medical treatment alone. Such high proportions may reflect national guidelines that encourage utilization of non-medical lifestyle treatments in patients below recommended thresholds for initiation of medical treatments. But they may also in part reflect the reliance on non-medical therapies in a subgroup of patients who cannot afford or access medical treatments.

Although guidance on diagnostic thresholds for initiating medical treatment may vary, non-medical interventions are recommended in all individuals at the diagnostic threshold used in this study [33,34]. In Mongolia 14% of hypertensives reported receiving medical, but no non-medical treatments raising concerns about clinical practice. It appears that appropriate lifestyle advice that can improve management is not reaching many of these people.

The results suggest that diagnosis or initiation of treatment does not ensure effective disease management. Point estimates indicate worrisome levels of uncontrolled blood pressure and blood glucose among people on treatment and poor life-style advice levels amongst subgroups especially in need.

### Implications for service development

Our findings for diagnosis gap and treatment coverage and quality are in line with other research that has drawn attention to suboptimal diagnosis and management of NCD risk factors. For example, some 70% of diabetics in seven countries in Europe, Asia and North and South America were assessed as failing to attain recommended blood sugar levels [12,13]. To reduce CVD mortality, a key goal of primary prevention strategies is to achieve a reasonable level of control of these risk factors. For example, one of the national objectives under Healthy People 2010 initiative in USA includes controlling blood pressure in 50% of all individuals with hypertension [10].

This study suggests that in Cambodia and Mongolia only about a quarter of all hypertension and less than a quarter of all diabetes is adequately controlled. Thus improvement in primary prevention strategies will require multifaceted efforts to improve detection and diagnosis, treatment of those diagnosed, and the quality of treatment among those who are treated In addition, our findings suggest improvements can be made in optimal use of non-medical therapies.

Finally, the very high prevalence of risk factors as defined at the cut-offs levels used for each risk factor may imply need for substantial increase in health financing to provide drug treatment. Global absolute CVD risk assessment based on levels of multiple risk factors may be used to prioritize people with higher overall CVD risk for drug treatment, while those with lower overall risk may be followed-up with systematic life-style interventions. WHO and International Society for Hypertension has published cardiovascular risk prediction charts for all the fourteen WHO epidemiological sub-regions that can be used by physicians or non-physicians in low-resource settings to estimate 10-year risk of a fatal or non-fatal cardiovascular event [33]. Recent research has suggested this approach offers substantial cost-saving in drug treatments [35]. Appropriate screening strategies will still be required for early identification of people with moderate to high absolute global CVD risk, but to date none of the countries have formally introduced clinical guidelines either for screening or treatment based on absolute CVD risk assessment, though Mongolia is in the process of developing these guidelines.

### Limitations

The study has limitations that must be acknowledged. For example, due to the surveys' cross-sectional nature, whereas guidelines recommend that diagnosis should only be made on the basis of raised blood pressure sustained over time, the study diagnoses of hypertension are based upon multiple measurements at a single visit and thus may not be sufficient for a confirmed diagnosis or to assess the adequacy of control in individuals [16]. There may be inter-investigator variations in measurement that were not assessed in the survey and hence were not taken into account. The advice to refrain from the taking of diabetes medications on the morning of the blood tests may have delayed medication for some hours and affected readings in some participants. Adherence these and to fasting instructions is unknown. While the literature is consistent on suitability of capillary whole blood for screening of diabetes in population based surveys, the cut-off used to diagnose diabetes for capillary blood versus venous plasma is still debatable [23,36]. In the absence of any clear national guidelines in either of these countries, we used the most-commonly used cut-offs reported in international literature and guidelines. Measures of prevalence, diagnosis and treatment gap will vary greatly based on the diagnostic cut-off for hypertension and diabetes,, though this methodology will still be appropriate to assess the trends in these indicators over time if similar measurement methods and diagnostic cut-offs are used. Finally, the study does not capture the complete disease burden that health systems may face, due to exclusion of population aged over 65, which may have much higher disease burden.

### Implications for future surveys/research to monitor response to NCDs

With regards to self-report of previous testing, it was not possible to ascertain whether individuals were tested previously as part of formal, opportunistic or universal screening programs or were simply tested when they sought services on experiencing symptoms. Nor can the source of testing be discerned: whether public or private or whether in primary or secondary care. Inclusion of questions on context of testing in future surveys may help to evaluate early detection strategies as more countries scale up NCD early diagnosis and care programs. Additional questions on adherence to treatment, cost and affordability of treatment and source of treatment may be included to distinguish and estimate the demand and supply-side factors affecting the treatment coverage. We were unable to examine the primary prevention of hypercholesterolemia due to lack of medical history for prior diagnosis and treatment. Considering the prominence given to high cholesterol levels in determining absolute CVD risk, the medical history for hypercholesterolemia may be included in future surveys.

## Conclusions

The prevalence of risk factors for CVD is already at alarming levels in both countries. Only a small proportion of the total hypertensive or diabetic populations have adequately controlled blood pressure or blood sugar due to large diagnosis gap, non-treatment of previously diagnosed populations and inadequate control of treated population. Aggressive population-wide strategies complemented by individual-based primary prevention strategies will be required. Guidelines for concurrent screening of multiple risk factors in targeted population groups as identified based on epidemiological profile, which can be realistically delivered by health systems in these poorly resourced setting will be needed. Prioritization of individuals based on global CVD risk for drug treatment may reduce the overall cost of treatment. Non-medical treatment needs to be emphasized equally. Periodic population-wide surveys including questions on adherence to the treatment, reasons for not-seeking treatment, source and cost of the treatment, etc. should be conducted to better inform the programs and to monitor the performance of these efforts.

## Competing interests

The authors declare that they have no competing interests.

## Authors' contributions

MR and BB drafted the manuscript. EP and OD were the leading researchers for the STEPS survey in Mongolia and contributed to the conceptualization of the paper and writing. SO was the lead researcher for organization of STEPS survey in Cambodia, and contributed to the conceptualization of paper and writing. MR and BB carried out statistical analysis of the paper. All authors read, commented, and approved the final version of the manuscript.

## Authors' information

EP -Public Health Institute, MOH, Mongolia

SO - University of Health Sciences, Cambodia

OD - Public Health Institute, MOH, Mongolia

MR - Western Pacific Regional Office, World Health Organization

BB - University of the Philippines Manila, Philippines

## Pre-publication history

The pre-publication history for this paper can be accessed here:

http://www.biomedcentral.com/1471-2458/12/254/prepub
